# DISMANTLING AGEISM: EXPLORING PERSONAL AND SOCIETAL ATTITUDES ABOUT AGING IN UNDERGRADUATE CLASS ACTIVITIES

**DOI:** 10.1093/geroni/igac059.1713

**Published:** 2022-12-20

**Authors:** Erica Srinivasan

**Affiliations:** University of Wisconsin, La Crosse, La Crosse, Wisconsin, United States

## Abstract

Ageism and fear of aging are influenced by both personal and societal attitudes about aging. Ageist beliefs reflected in society or in individuals can impact both our own aging process and how we treat others (Cooney et al, 2020). Thus, it is imperative to explore personal and societal attitudes about aging. In this poster, themes will be presented from a study on two class activities that explore ageism and attitudes about aging, as well as directions for these activities. Students (n=34) in an undergraduate college class were asked to describe their thoughts on aging in 6 words. They were next prompted, with no word limit, to describe fears and what they look forward to about aging. Content analysis of responses revealed themes including the complexity of aging, fears about dementia, physical changes, and the unknown nature of life, and positive interest in career and family goals. In a second activity, based off the AARP social experiment in which millennials were asked “what do you consider to be old?,” students asked peers “what image comes to mind when I say “old person?” and “what three words come to mind when I say old person?” Content analysis of responses included themes relating primarily to negative stereotypes, such as “confused and nursing home,” with some positive associations, such as “grandparents or wisdom.” Both activities prompted rich discussion about the connection between fears of aging and ageism, the importance of addressing ageism and of exploring both positive and challenging aspects of personal aging.

